# Mental Symptoms and Stress of Hospitalized Schizophrenia Patients With 2019 Novel Coronavirus Disease: An Observation Study

**DOI:** 10.3389/fpsyt.2021.557611

**Published:** 2021-04-09

**Authors:** Jun Ma, Tao Jiang, Hanjun Huang, Ruihua Li, Lin Zhang, Lianzhong Liu, Xuebing Liu

**Affiliations:** ^1^Affiliated Wuhan Mental Health Center, Tongji Medical College of Huazhong University of Science & Technology, Wuhan, China; ^2^Shanghai Mental Health Center, Shanghai Jiao Tong University School of Medicine, Shanghai, China

**Keywords:** COVID-19, schizophrenia, psychopathology, stress, medical isolation

## Abstract

**Background:** The 2019 novel coronavirus disease (COVID-19) is an extremely rapidly spreading respiratory infection caused by SARS-CoV-2. Many schizophrenic patients were infected with COVID-19 in Wuhan City, Hubei Province. This study took hospitalized schizophrenia patients with COVID-19 as the research subjects and observed the changes in psychopathology and stress of patients with COVID-19 and the accompanying social isolation.

**Methods:** To sort and isolate potential COVID-19-infected patients, an isolated ward was set up from January 30, 2020, to March 30, 2020. Schizophrenia patients with COVID-19 were referred to this ward, and long-term hospitalized cases were included in this study. The Positive and Negative Syndrome Scale and Perceived Stress Scale were used to evaluate the severity of mental symptoms and psychological stress in the early stage of the outbreak of COVID-19, after the diagnosis of COVID-19 and after recovery. At the time of diagnosis, we also extracted the patient's routine blood, biochemical and other indicators and asked the patient's perception of COVID-19.

**Results:** 21 hospitalized schizophrenia patients with COVID-19 were recruited in this study. The changes in PANSS scores were not significant (*p* = 0.225 baseline *vs*. diagnosed, *p* = 0.399 cured *vs*. diagnosed). The CPSS scores increased significantly after diagnosis and transfer to the isolation ward (*p* < 0.001 baseline *vs*. diagnosed, *p* < 0.001 cured *vs*. diagnosed). The course of schizophrenia was a protective factor of stress levels to cases (*t* = −3.25, *p* = 0.006), and patients' perception of COVID-19 was a risk factor (*t* = 2.48, *p* = 0.038). The final multiple linear regression model was statistically significant (*F* = 8.16, *p* < 0.001).

**Conclusion:** Hospitalized schizophrenia patients with COVID-19 had increased stress levels and negative symptoms but alleviated positive symptoms after medical isolated treatment. This reminds us that in the face of major epidemics, we must specifically alleviate the psychological burden at the peak of the epidemic and improve the prognosis of patients after the epidemic.

## Introduction

The 2019 novel coronavirus disease (COVID-19) is an acute respiratory infectious disease caused by the new coronavirus (SARS-CoV-2) (1). In Wuhan, Hubei province, the actual number of SARS-COV-2 infection cases might be much higher than that has been reported (2). The rapid spread of the COVID-19 and its serious consequences pose severe challenges to public health in China and around the world (3, 4).

Since the outbreak of COVID-19, researchers have made numerous reports on the epidemiological characteristics, clinical characteristics, and prognosis of infection cases (5–8). As a stressful event with unknown treatment efficacy, prognosis, and mortality in the early stage of the epidemic, the population exposed to COVID-19 faced the severe challenge of psychological tolerance. According to a recent report by Wang et al. (9), in the early stage of the COVID-19 outbreak, more than half of the respondents in the general population were rated as moderate-to-severe in their psychological impact, and approximately one-third exhibited moderate-to-severe anxiety. Meanwhile, Hao et al. also confirmed that the strict lockdown measures accompanied by COVID-19 have a serious negative impact on psychiatric patients (10). A recent Italian study also reported that the COVID-19 pandemic has brought negative emotions to patients with bipolar disorder (11). For mental patients, stressful events are important factors that aggravate mental symptoms (12, 13).

Owing to the highly contagious nature of viruses, a considerable number of severe mental patients in Wuhan City, Hubei Province, have not been spared, including long-term hospitalized schizophrenic patients. However, due to the special management of mental diseases and requirements for epidemic prevention and control, few researchers have studied the effects of COVID-19 on the mental symptoms of severe psychotic patients. Hence, we examined schizophrenia patients with COVID-19 in a psychiatric specialty hospital located in Wuhan, Hubei Province, and conducted a study on the effect of COVID-19 and the accompanying social isolation on psychopathology and stress.

## Methods

### Subjects

We collected long-term hospitalized psychiatric patients who were required to meet the diagnostic criteria of schizophrenia in the Diagnostic and Statistical Manual of Mental Disorders, Fifth Edition (DSM-5). They were between 20 and 65 years old, had stayed in the hospital for more than 2 years, and had hospital-acquired infections and were diagnosed with COVID-19. Polymerase chain reaction (PCR) testing for SARS-CoV-2 was positive, and chest CT scans showed patchy or frosted ground glass-like images, regardless of sex, unrestricted type, and measurement of antipsychotic drugs, and whether it was accompanied by common physical diseases such as hypertension, diabetes, and hyperlipidemia.

Patients with bipolar disorder, substance dependence, personality disorder, intellectual developmental disorder, severe cognitive impairment, and mental disorders caused by physical diseases were excluded. In addition, critically ill patients with blood oxygen saturation below 93%, dyspnea, and patients with unstable vital signs were excluded. These patients were all transferred to designated hospitals for the treatment of severe diseases. Patients who were unable to cooperate with isolation treatment, such as severe violence and suicide, were also excluded, and these patients were transferred to special wards with a dedicated work team for intervention.

This study was reviewed and approved by the ethics committee of the medical institution where the patients were housed. All enrolled patients received written consent from the patient's family.

### Instruments

The electronic medical records were used to extract clinical characteristics, chest imaging characteristics, blood convention, biochemical indicators, and C-reactive protein (CRP) of the patients. The Positive and Negative Symptom Scale (PANSS) was used to assess the psychopathological changes of the included patients, and the Chinese Perceived Stress Scale (CPSS) was used to assess patients' perception of stress. According to PANSS's classification of mental symptoms, PANSS has three subscales: PANSS positive symptom subscale (PSS, items P1-7), PANSS negative symptom subscale (NSS, items N1-7), and PANSS General Psychopathology scale (GPS, items G1-16).

We added seven additional questions to assess the perception of COVID-19 in the included cases (called the COVID-19 perception questionnaire, CPQ), which were as follows:

1. Have you heard of the 2019 novel coronavirus disease?
2. Options setting: yes and no
3. Do you know that the patients around you are also infected with this disease?
4. Options setting: yes and no
5. Do you know why you transferred to this ward?
6. Options setting: yes and no
7. Are you worried about the treatment effect after infection?
8. Options setting: very worried, general, not worried
9. Are you worried about the dress of medical workers?
10. Options setting: very worried, general, not worried
11. Are you worried about your family being infected?
12. Options setting: very worried, general, not worried
13. Do you know the epidemic trend of the 2019 novel coronavirus disease?
14. Options setting: yes and no

Scoring principle: We mark “yes” and “generally” equal to 1 point, “no” or “not worried” equal to 0 points, and “very worried” equal to two points. The higher the total score, the more comprehensive the patient's perception of COVID-19, and vice versa.

### Procedures

This study was designed as a clinical observation study. As early as January 2020, when the epidemic had not been reported on a large scale, we completed the initial assessment of PANSS and CPSS for the long-term hospitalized schizophrenic patients. At that time (baseline), the patients were uninfected. As the epidemic continued to spread, isolation wards were set up in a certain psychiatric institution in Wuhan, Hubei Province, China on January 30, 2020, for the isolation and treatment of psychotic patients who were diagnosed with or suspected to have COVID-19. The isolation ward is set up ranging from 1 to 3 patients per room. After a week's work of ward reconstruction, the admission of patients began. From then on, the cured cases would be transferred out of the isolation ward, and at the same time, newly infected patients would be transferred into the isolation ward. We selected the confirmed cases of COVID-19, from transferred to the isolation ward to cured and transferred out for further follow-up observation. All medical services followed the Diagnosis and treatment of corona virus disease-19 issued by the National Health Commission of China. According to the guidelines, the drugs taken are antiviral, anti-inflammatory, and Chinese patent medicine. The second PANSS and CPSS were estimated within 3 days of diagnosis after the patient was transferred to the isolation ward. At this time, the routine blood, C-reactive protein, and biochemical indexes of the patient were extracted from the electronic medical record and CPQ were estimated. After the cases were cured, before they were transferred out of the isolation ward, the third PANSS and CPSS evaluation were performed. The raters in this study were all psychiatrists with professional training and experience in managing psychopathological tests.

We used a table to show the detailed characteristics of the general clinical data and longitudinally compared the differences in the total score of PANSS, the scores of the three subscales of PANSS and CPSS at different time points. We also analyzed the factors affecting the stress levels of the included patients.

### Data Analysis

According to the characteristics of the final data, if the continuous measurement data obtained were normally distributed, they were expressed as the mean and standard deviation (SD). The categorical variables were expressed as counts and percentages. Paired *t*-test was performed on continuous variables with normal distribution, and multiple linear regression was used to analyze stress factors. The significance level of all statistical tests was set as *p* < 0.05 (two tails). Data analysis was performed using IBM SPSS version 26.0 statistical software (SPSS Inc., Chicago, IL, USA), and figures were plotted using GraphPad Prism version 8.4 software (GraphPad Software Inc., La Jolla, CA, USA).

## Results

### General Clinical Treatment Characteristics

A total of 57 patients entered the ward for screening because of suspected or confirmed COVID-19, and 21 schizophrenic patients with COVID-19 were cured and discharged. Following the requirements of epidemic control, the transfer of the last cured patient to the designated site for continued observation was completed at 6 p.m. on 30 March 2020.

The general clinical characteristics of the 21 patients who completed this study are shown in Table 1.

**Table 1:** Clinical characteristics of schizophrenia patients with COVID-19.

**Index**	**COVID-19 patients (*n* = 21)**
**Age** - **years**	
Mean (SD)	43.1 (2.6)
Range	24–61
**Sex**	
Female	12 (57.1%)
Male	9 (42.9%)
Length of stay – years (SD)	4.2 (3.4)
Course of schizophrenia-years (SD)	6.8 (5.6)
Duration of healing – days (SD)	31 (10.2)
Take an antipsychotic drug- *n* (%)	5 (23.8%)
Take two antipsychotic drugs- *n* (%)	16 (76.2%)
Protective constraint – *n*	0
**Infection symptoms**	
Asymptomatic throughout	4 (23.8%)
Respiratory symptoms	8 (38.1%)
Digestive symptoms (diarrhea)	1 (4.8%)
Only one symptom of fever	8 (38.1%)
**First chest CT findings -** ***n*** **(%)**	
Unilateral	10 (47.6%)
Bilateral	11 (52.4%)
**Worsening infection following chest CT reexamination**	
Yes	16 (76.2%)
No	5 (23.8%)
**Routine blood test** - ***n*** **(%)**	
Leukopenia	4 (19.0%)
Lymphocytopenia	6 (28.6%)
Both	11 (52.4%)
Elevated CRP	4 (19.0%)
Normal CRP	17 (81.0%)
Additional intravenous antibiotic therapy	4 (19.0%)
**Adjustment of antipsychotic drugs during isolation**	
Increase in the doses	1 (0.5%)
Increased benzodiazepines	2 (1%)
Decrease in the doses	2 (1%)

### The Difference of the PANSS and Its Subscales and CPSS

**Figure 1:**
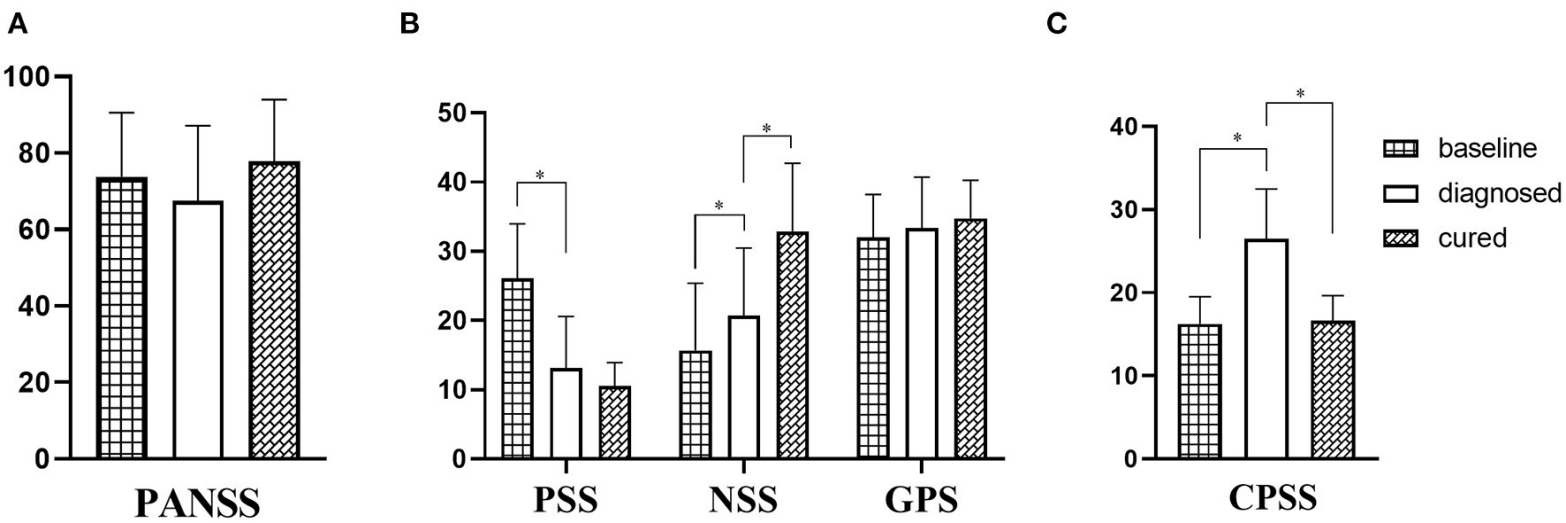
**(A)** Comparison of PANSS scores at three time points. There is no statistical difference in PANSS score at different time. **(B)** Comparison of PANSS subscale scores at three time points. The PSS score was significantly lower at the time of diagnosis than at the baseline. The NSS score at diagnosed is significantly higher than at baseline and significantly lower than at cured. There is no difference in GPS scores at different time. **(C)** Comparison of CPSS scores at three time points. The CPSS score at the time of diagnosed is significantly higher than that at baseline and cured.

There were no significant differences in PANSS scores of the included patients at the three time points of the early stage of the epidemic (baseline), within 3 days after diagnosis with COVID-19 and transported to the isolation ward (diagnosed) and cured (cured) (*p* = 0.225 baseline *vs*. diagnosed, *p* = 0.399 cured vs. diagnosed) (Figure 1A). In addition, the positive symptom subscale scores of “diagnosed” were significantly lower than those of “baseline” (*p* < *0.001*) (Figure 1B). The negative symptom subscale scores were significantly higher when “diagnosed” compared to “baseline” (*p* < *0.001*) and “cured” compared to “diagnosed” (*p* < *0.001*). The CPSS scores of “diagnosed” patients were significantly higher than those of “baseline” (*p* < *0.001*) and “cured” (*p* < 0.001) (Figure 1C).

### Multiple Linear Regression Analysis of Influencing Factors of Patients' Psychological Stress

Multiple linear regression analysis was used to analyze the psychological pressure of patients (CPSS) when diagnosed as a dependent variable, and gender, age, course of schizophrenia, duration of hospitalization, years of education, symptoms associated with infection and COVID-19 perception questionnaire (CPQ) were independent variables, as shown in Table 2. The course of schizophrenia was a protective factor of stress levels to cases (*p* = 0.006), and patients' perception of COVID-19 was a risk factor (*p* = 0.038). The final multiple linear regression model was statistically significant (*F* = 8.16, *p* < *0.001*).

**Table 2:** Multiple linear regression analysis of influencing factors of patients' psychological stress.

**Variable**	**Coefficient**	**Standard** **deviation**	**Standardized** **coefficients**	**95% CI**	***t***	***p***
Constant	41.18	7.47		(25.16, 57.20)	5.51	0.000
Sex (female vs. male)	−4.77	2.73	−0.40	(−10.62, 1.08)	−1.75	0.102
Age	0.06	0.13	0.11	(−0.23, 0.35)	0.42	0.684
Course of schizophrenia	−0.49	0.15	−0.81	(−0.82, 0.17)	−3.25	0.006^*^
Duration of hospitalization	−0.14	0.48	−0.06	(−1.17,0.89)	−0.29	0.774
Years of education	−0.40	0.33	−0.21	(−1.11,0.32)	−1.19	0.254
Symptoms associated with infection (without *vs*. with)	5.77	3.05	0.39	(−0.77, 12.31)	1.89	0.079
CPQ	2.08	0.84	0.66	(2.65, 3.91)	2.48	0.038^*^

* *p < 0.05 (CPQ, COVID-19 perception questionnaire, Mean ± standard deviation: 6.19 ± 1.91; 95% CI, 95% Confidence Interval)*.

## Discussion

To the best of our knowledge, this is the first clinical study on schizophrenia patients with COVID-19, investigating the changes in psychological pressure and psychiatric symptoms in cases with COVID-19 infection and isolation therapy. We found that patients with COVID-19 did experience increased stress levels and negative symptoms but alleviated positive symptoms. The course of schizophrenia was a protective factor of stress levels in cases; in contrast, patients' perception of COVID-19 was a risk factor.

As a global pandemic impacting public safety, the general population is susceptible to COVID-19. The rapid spread of the virus and the uncertainty of the virus significantly increased the psychological burden of the general population (9). People with stable clinical symptoms after COVID-19 infection also showed obvious symptoms of posttraumatic stress (14). Another study recently studied people who developed psychiatric symptoms and found that psychotic episodes were significantly associated with coronavirus exposure (15). The latest research suggests that more than one-third of psychiatric patients might fulfill the diagnostic criteria post-traumatic stress disorder (PTSD) during the peak of COVID-19 epidemic with strict lockdown measures (10). Another study from the United Kingdom showed that those who have or had COVID-19-related symptoms are more likely to develop general psychiatric disorders (16). In addition, the general population without a history of psychiatry also showed psychotic symptoms with structured delusions mixed with confusion as a common feature after being infected with COVID-19 (17). However, none of these studies could specifically target schizophrenia patients with COVID-19. SARS-CoV-2, a novel coronavirus with similar neurotrophic effects (18), was only reported for the first time after the outbreak. To date, we have not had enough time to track the future incidence of mental disease of nonpsychotic patients who were exposed to COVID-19 and the long-term impact of psychiatric symptoms on schizophrenic patients exposed to COVID-19. As far as this study, there were no significant changes in psychiatric symptoms in schizophrenia patients with COVID-19 during short-term follow-up observations. However, this is not the end, as COVID-19 may have a long-term impact on the mentally ill, and we will perform in-depth follow-up observations.

Regardless of the effect of nervous system infection on mental illness, stressful events have always been considered one of the important factors for the occurrence and deterioration of mental illness (12, 13). An interaction between external stressors and intrinsic vulnerability was one of the longest standing pathoaetiological explanations for schizophrenia, also known as Diathesis-Stress Hypothesis (19). The hypothesis suggest that psychosocial stress may promote pathological microglia activation, which may lead to excessive synapse pruning and loss of cortical gray matter. Based on this, if the stress-sensitive area is damaged, this may lead directly to cognitive and negative symptoms; and loss of cortical control may also lead to disinhibition of subcortical dopamine—thereby leading to positive psychotic symptoms.

In this study, we confirmed that COVID-19 and transfer to isolation wards significantly increased the perception of psychological pressure. However, the increase in psychological pressure may not only be limited to COVID-19 itself, which also includes the strict isolation measures that have led to more narrow living spaces and the impact of environmental changes such as the different dress of the medical staff during wards rounds. Analysis of the factors that affect patients' psychological stress found that the course of schizophrenia constitutes an independent influencing factor for the reduction in psychological stress, that is, the longer the course of illness, the less psychological stress the patient would feel. A 6-year follow-up study found that the cognitive function of long-term hospitalized schizophrenia patients will gradually deteriorate over time (20). Based on this, we infer that the cognitive capacity of the enrolled patients decreased significantly due to the longer course of schizophrenia, thereby reducing the patients' awareness of the threat and danger of COVID-19, and thus reducing the psychological stress they presented. It should be emphasized that this psychological stress only manifested in the early stage of diagnosis of COVID-19, and the adaptation to the environment after a longer period of isolation may alleviate the patient's perception of stress.

Our study found that the severity of psychiatric symptoms of patients during isolation did not change significantly, which corresponds with the fact that the dosage of antipsychotic drugs is rarely adjusted during isolation therapy. This is different from the results of previous studies (21–23), which supposed stress and the severity of psychiatric symptoms were significantly related. However, without exception, all the previous studies took daily stress events as research elements. These stress events are more moderate in intensity, longer in time, and less threatening. This is the biggest difference between daily stress events and the stress events of this epidemic and may be the main reason for the difference in research results. This is the biggest difference between daily stress events and stressful events in the context of this epidemic. This may also be the main reason for the difference in research results. Additionally, positive symptoms were reduced, while negative symptoms were increased during the study. An animal study showed that socially isolated mice exhibited schizophrenia-like behaviors, such as a negative symptom phenotype (24). We speculated that the deterioration of negative symptoms in patients was the result of long severe social isolation and lack of adequate interpersonal communication caused by the COVID-19.

This article also has certain deficiencies. Due to the need for epidemic prevention and control, the patients in the group were transferred to the designated place for further isolation and observation after reaching the discharge standard. Therefore, our observation of the patients is short-lived, and the long-term impact of COVID-19 infection on schizophrenic patients needs further follow-up observation. To prevent cross-infection, it is difficult to carry out a larger sample study at present. A smaller sample size also harms the statistical efficiency of the study.

In conclusion, hospitalized schizophrenia patients with COVID-19 had increased stress levels and negative symptoms but alleviated positive symptoms after medical isolated treatment. This suggests that effective measures should be taken to relieve the psychological pressure of exposing patients with schizophrenia during the outbreak of a major epidemic, and targeted relief of negative symptoms of the patients is needed after the epidemic.

## Data Availability Statement

The raw data supporting the conclusions of this article will be made available by the authors, without undue reservation.

## Ethics Statement

The studies involving human participants were reviewed and approved by The Ethics Committee of Wuhan Mental Health Center is affiliated to Wuhan Mental Health Center. The patients/participants provided their written informed consent to participate in this study.

## Author Contributions

LL and XL made substantial contributions to conception and design of the study. LZ and RL collected and collated the data. JM analyzed and interpreted data and drafted and revised the manuscript. JM and TJ were responsible for the evaluation of the scale. HH collected imaging data. XL gave final approval of the version to be published. All authors contributed to the article and approved the submitted version.

## Conflict of Interest

The authors declare that the research was conducted in the absence of any commercial or financial relationships that could be construed as a potential conflict of interest.
